# Can Mindfulness-Based Training Improve Positive Emotion and Cognitive Ability in Chinese Non-clinical Population? A Pilot Study

**DOI:** 10.3389/fpsyg.2019.01549

**Published:** 2019-07-03

**Authors:** Tingfei Zhu, Jiang Xue, Astrid Montuclard, Yuxing Jiang, Wenqi Weng, Shulin Chen

**Affiliations:** ^1^Department of Psychology and Behavioral Sciences, Zhejiang University, Hangzhou, China; ^2^Department of Human Ecology, University of California, Davis, Davis, CA, United States

**Keywords:** mindfulness-based training, higher education students, non-clinical population, emotion, sustained attention, paired controlled design

## Abstract

**Objective:**

Based on eastern philosophy, mindfulness is becoming popular for human being’s mental health and well-being in western countries. In this study, we proposed to explore the effectiveness and potential pathway of mindfulness-based training (MBT) on Chinese Non-clinical higher education students’ cognition and emotion.

**Methods:**

A paired control design was used. 48 higher education students (24 in MBT group, 24 in control group) were recruited in the study. The MBT group engaged in a 12-week MBT. A package of measurements, including sustained attention tasks (The Continuous Performance Test, CPT), executive function task (Stroop) for cognitive functions, the self-reported mindfulness levels (The Mindful Attention Awareness Scale, MAAS) and emotion (The Profile of Mood States, POMS), were apply for all participants at baseline and every 4 weeks during next 12 weeks.

**Results:**

There were no differences in baseline demographic variables between two groups. Over the 12-week training, participants assigned to MBT group had a significantly greater reduction in CPT reaction time (Cohen’s d 0.72), significantly greater improvement in positive emotion (Vigor-Activity, VA) (Cohen’s d 1.08) and in MAAS (Cohen’s d 0.49) than those assigned to control group. And, MAAS at 4th week could significantly predict the CPT RT and VA at 8th week in the MBT group. VA at 4th week could significantly predict the CPT RT at 8th week (*B* = 4.88, *t =* 2.21, *p =* 0.034, *R^2^*= 0.35).

**Conclusion:**

This study shows the efficiency of 12-week MBT on Chinese Non-clinical students’ cognition and emotion. Mindfulness training may impact cognition and emotion through the improvement in mindfulness level, and may impact cognition through the improvement in positive emotion.

## Introduction

Mindfulness practice, which initially arose 2,500 years ago in eastern countries within the spiritual context of Buddhism (Kabatzinn, 2003), was systematically and scientifically examined by western scholars. Over the past decades, mindfulness practice has become quite popular in various research areas, such as psychology (Chambers et al., 2009; Chiesa et al., 2011), psychiatry (Chiesa and Serretti, 2011; Am et al., 2015), immunology (Chambers and Schauenstein, 2000), and neuroscience (Tang and Posner, 2015; Wheeler et al., 2017). The benefits of mindfulness-based trainings (MBTs) on physical, psychological and social functions have been well documented in western nations (Chiesa et al., 2011; Hülsheger et al., 2013). Mindfulness is commonly defined as “paying attention in a particular way; on purpose, in the present moment, and Non-judgmentally” (Kabatzinn, 2003). To some extent, “paying attention on purpose” coincides with sustained attention, “in the present moment” coincides with executive function, and “Non-judgmentally” coincides with emotion regulation. Thus, the definition’s keywords point to cognitive and emotional capacity training.

A growing body of robust evidence from researches has demonstrated that MBTs are effective in improving a range of cognitive outcomes in comparison to control conditions, including sustained attention (Jha et al., 2007; Tang et al., 2007; Lutz et al., 2009; Schmertz et al., 2009; Zeidan et al., 2010) and executive functions (Diamond and Lee, 2011; Tang et al., 2012; Moynihan, 2013; Lyvers et al., 2014). And a systematic review of neuropsychological findings demonstrated the effect of MBT on cognitive abilities (Chiesa et al., 2011). In Chambers’ study, the MBT group’s overall reaction times (RTs) of the internal switching task significantly improved from T_1_ to T_2_, whereas the controls’ did not (Chambers et al., 2008).

Mindfulness have been shown to be effective in improving emotional well-being. In China, rapid urbanization and economic growth continue to negatively impact public mental health (Gong et al., 2012; Zhong et al., 2017). Meanwhile, both subjective self-reports and objective measurements of physiological indicators have indicated that MBT can decrease the intensity of emotional responses’ negativity (Arch and Craske, 2006) and increase emotional regulation ability (Chambers et al., 2015; Wheeler et al., 2017). Mindfulness significantly mediated the effects of MBTs on mental health outcomes, including anxiety, mood states, negative affect and depressive symptoms (*z* = 4.99, *SE* = 0.02, *p <* 0.001) in previous studies (Gu et al., 2015). Those results were even observed in patients with conditions such as Generalized Anxiety Disorder or depression (Goldin and Gross, 2010; Chambers et al., 2015; Xu et al., 2015).

Although MBT has shown consistent efficacy for many physical and mental disorders in western country, less attention has been given to the possible benefits and feasibility that it may have in Non-clinical population, especially in China (Tang et al., 2007, 2009), the most populous eastern country in the world. Application of MBT might take effect in Non-clinical population as a tool for the reduction of stress, the improvement of quality of life and prevention of mental disorders. Continuous stress may lead to unproductive rumination that consumes energy and strengthens the experience of stress itself (Trapnell and Campbell, 1999), and too much stress can adversely affect emotion (Allen et al., 2014) and cognition (Liston et al., 2009; Wu et al., 2014). However, several reviews were found about MBT in Non-clinical population. In the review about Mindfulness-Based Stress Reduction (MBSR) for Non-clinical individuals in 2014 (Manoj and Rush, 2014), only 17 articles met the inclusion criteria and none of them focused on Chines. A meta-analysis in 2009 found only ten studies published before 2008 that focused on Non-clinical population (Chiesa and Serretti, 2009), and still none of these researches focused on Chinese volunteers.

Besides, mindfulness would be a more economical way to support young people’s health, while participation in higher education is growing among young people. 45.7% of young people now enter higher education in Ministry of Education of the People’s Republic of China (2018). Prevalence of mental illness in undergraduates becomes higher after first-year study (Macaskill, 2013). Thus, the higher education journey provides a golden opportunity for prevention of mental illness in young people.

In addition, although many research studies demonstrated in the west that mindfulness can reduce anxiety, depression (Hoge et al., 2013; Würtzen et al., 2013), insomnia (Ong et al., 2014), and relieve chronic diseases (Bohlmeijer et al., 2010), few studies have focused on the mechanisms of mindfulness and related studies only appeared in recent years (Bailey et al., 2016; Brake et al., 2016; Alsubaie et al., 2017; Lindsay and Creswell, 2017), and the underlying mechanism for mindfulness remains unclear.

Emotion and cognition improvements are commonly known as the outcomes of MBT; however, the roles they played during MBT were rarely explored. The relationship between emotion and cognition becomes a focus of research recently. In the spatial orienting tasks, where there is a faster response to targets appearing on the same side as an emotional cue (e.g., faces, spiders) and a slower response to those appearing on the opposite side (Mogg et al., 1997; Armony and Dolan, 2002). Anatomically, the amygdala (modulate the function of regions involved in early object perceptual processing) receives visual inputs from ventral visual pathways and sends feedback projections to all processing stages within this pathway (Eslinger, 1992). This finding may explain how pre-attentive processing of emotional events influences and enhances perception. Besides, Researches demonstrated that MBTs are effective in retrieval of specific autobiographical memories (Williams et al., 2000), a reliable cognitive marker of depression (Brittlebank et al., 1993). All these findings have shown that there might be a potential pathway of influencing cognition through emotion (Dolan, 2002).

In light of existing research literatures and current research needs, the present study attempted to: (a) determine the effectiveness of a 12-week MBT in Chinese Non-clinical population; (b) explore the potential pathway of mindfulness training through the relationship between the mindfulness level and cognition/emotion; (c) explore the potential pathway of mindfulness training through the relationship between the emotion and cognition.

We hypothesized that: (a) the 12-week MBT would affect Chinese Non-clinical higher education students’ cognition and emotion, resulting in a significant increase in sustained attention, executive function and positive emotion, as well as decrease in negative emotion; (b) mindfulness training may take effect through the potential pathway of influencing cognition and emotion through the improvement in mindfulness level; (c) mindfulness training may take effect through the potential pathway of influencing cognition through the improvement in emotion.

## Materials and Methods

### Participants and Procedures

A paired control trial was used in this study. The study was conducted aiming to investigate the effect of a MBT program compared with a control group. Participants were considered eligible if they met the following criteria: (1) have no practice of any form of MBT; (2) over 18 years of age; (3) higher education student in Zhejiang university; (4) ability to communicate independently and understand tasks; (5) willing to participate in the research and give informed consent. Exclusion criteria were: (1) severe neuropsychological impairment; (2) psychosis or dissociative disorders.

A total of 54 participants were recruited through a voluntary research participation of Zhejiang University in Hangzhou, China. All the 54 participants responded to the study announcement and all the participants interested by the study were included. Participants were matched by sex and age, after which the paired two participants were randomly assigned to mindfulness training group or control group. Randomization function in Excel 2016 was used and performed by the principal investigator prior to any contact with participants. Participants in MBT group were asked to complete 12-week MBT, and the participants in control group got brief poster about MBT. Follow-up assessments were scheduled with the participants and they were provided a reminder 1 week before. All participants completed the first assessment, and 48 of them completed the entire study (6 dropouts/11.1%). Six participants refused to participate in the follow-up study.

**FIGURE 1:**
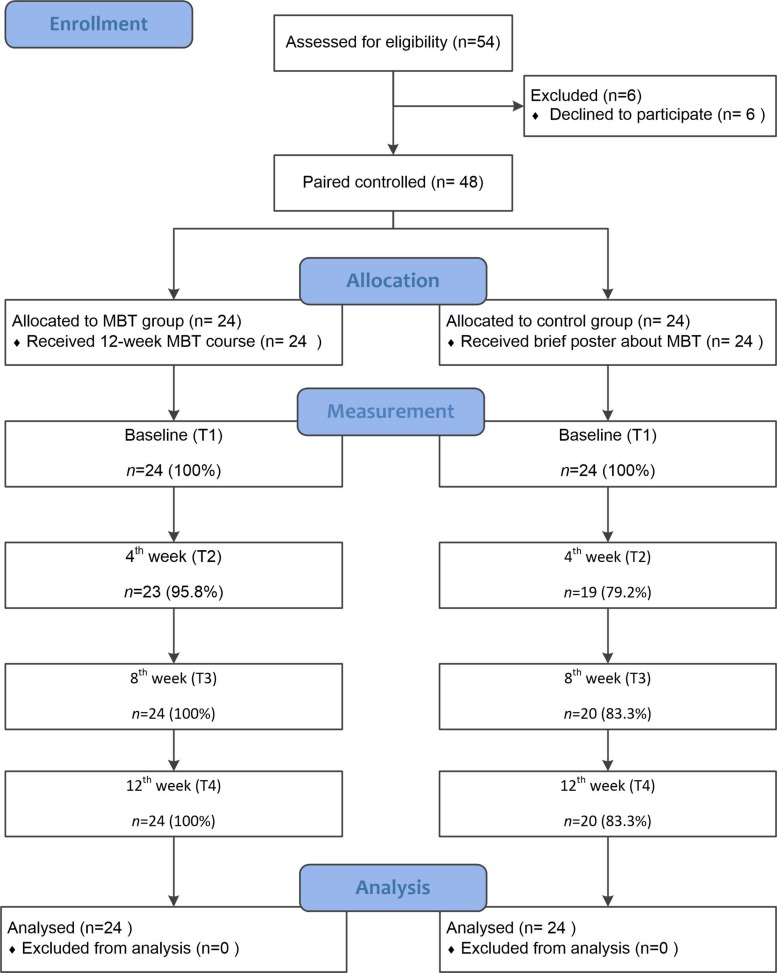
Research procedures.

The training lasted 12 weeks. The MBT course followed the original MBSR structure. Demographic data, together with information about mindfulness, emotion, sustained attention and executive function were collected. Participants were assessed at the recruitment baseline (T_1_), after 4-week training (T_2_), after 8-week training (T_3_) and after 12-week training (T_4_) (see Figure 1). The attendances of MBTs and measurements were recorded, indicating that all the absences were due to unavailable of schedule. Recruitment and assessment procedures were conducted in the Zhejiang university. This study was carried out in accordance with the recommendations of Institutional Review Board of Zhejiang University. All subjects gave written informed consent in accordance with the Declaration of Helsinki.

TZ enrolled participants and assigned participants to intervention, JX generated the allocation sequence.

### Mindfulness Training

The MBT delivered to the training group was modeled on Jon Kabat-Zinn’s MBSR Curriculum, as described on the Palouse Mindfulness website of Dr. Dave Potter, Certified MBSR Teacher. The training’s content was rooted in an academic 12-week mindfulness course’s curriculum designed by a University of Massachusetts Center for Mindfulness-certified teacher. All mindfulness lessons were conducted by the third author (AM), who had 2 years of personal mindfulness practice and 6 months of experience in leading a mindfulness continuing practice group. The facilitator did not have a certification in mindfulness teaching, but this situation is similar to the one of many teachers delivering mindfulness training in schools or online (Napoli et al., 2005; Mendelson et al., 2010; Cavanagh et al., 2013).

Table A1 outlines the content of the training. Exercises included mindfulness meditation practice (body scan, breath meditation, emotion and thought meditation…) as well as mindfulness skills (Recognize, Allow, Investigate, Non-identify (RAIN) technique…). Participants were encouraged to meditate at least 5 times a week between 10 and 20 min. The training group received MBT once a week and each session lasted about 1.5–2 h. Participation in sessions and out-of-class meditation was voluntary.

### Measures

#### Mindfulness

The Mindful Attention Awareness Scale (MAAS) was used as a dispositional measure of mindfulness. The MAAS is a 15-item scale assessing the frequency of mindful states in day-to-day life through the rating of both general and situation-specific statements. Higher scores indicated higher individual mindfulness level. The MAAS was shown to successfully report changes in mindfulness levels after MBT in several studies (Carlson and Brown, 2005; Shapiro et al., 2007). One previous study measured the MAAS’s Cronbach’s alpha level at 0.87 (Morone et al., 2016). The Chinese version revised by Chen also displays a high degree of validity with a Cronbach’s alpha level of 0.89 among Chinese participants (Chen et al., 2012). Cronbach’s alpha for MAAS in this study was 0.88.

#### Sustained Attention

The Continuous Performance Test (CPT) was used to assess sustained attention. CPT is a classical experimental paradigm for measuring sustained attention. The CPT has shown validity in different studies (Cornblatt et al., 1988; Berger and Cassuto, 2014; Junwon et al., 2015). In this task, participants were required to monitor a single letter of visual and to respond when a target stimulus occurred. The CPT was programmed in MATLAB utilizing a Lenovo LXM-L17AB computer. The task consisted of 11 letters (40 pt) flashing on the center of a video monitor (a 25.8 × 34.4 cm screen) for 130 ms at the rate of 600 ms between letters. The target was the letter X followed by the letter A. The task involved pressing the space key when the target appeared and avoiding responding to the other letter combinations (Klee and Garfinkel, 1983). CPT-AX task was chosen and included the capital letters A, B, C, D, E, F, G, H, J, L, X as stimulus. The participant is asked to respond as quickly as possible when the letter ‘X’ is followed by the letter ‘A’. True positives, false alarms and reaction time are recorded as dependent measures. There is a total of 20 target sequences out of a total of 960 trials. Each block contained 240 stimuli, and there were 4 blocks in total. In addition, participants followed a practice program before entering the formal experiment. Omission errors were scored for each target missed, while commission errors were scored for a response made to a Non-target stimulus (Klee and Garfinkel, 1983). Each experiment lasted for about 30 min.

#### Executive Functions

The Stroop is a classical experimental paradigm for measuring executive function, which was programmed in E-prime utilizing a Lenovo LXM-L17AB e computer with 17-inch color display and resolution of 1024 × 768. The refresh rate was 75 Hz. The stimulus were Chinese characters “
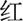
”, “
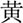
”, “
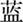
”, “
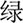
” (meaning “red”, “yellow”, “blue”, “green,” respectively). The stimuli were written in red, yellow, blue, green, respectively in congruent situation, and they were written randomly in red, yellow, blue, green in incongruent situation. The experiment consists of 4 blocks. Each block had 16 trials. The task involved judging the color of the stimulus and press the corresponding key: F for “red,” G for “yellow,” H for “blue” and J for “green.” Participants were asked to respond accurately and quickly. Participants first gazed to a cross ‘+’ for 800 ms, then the stimulus appeared for 150 ms, and 1750 ms were left for the participant to respond. A two-block practice program was available before the participant entered the formal experiment. An accuracy rate above 90% only allowed participants to enter the formal experiment. The experimental design balances variables such as consistency, color, prospective memory stimuli, and so on. The distance between the subjects and the monitor was about 60 cm, and all stimuli were presented in the center of the display screen, on a black background. The experiment was carried out in a soundproofed room and lasted for about 5 min.

#### Emotion

The Profile of Mood States -Short Form (POMS-SF) was used to measure emotion. The POMS-SF is a 30-item inventory measuring current mood state through statement ratings (e.g., I feel calm) on a Likert scale (0–4). The questionnaire consists of six subscales: “Tension-Anxiety (TA)”, “Depression-Dejection (DD)”, “Confusion-Bewilderment (CB)”, “Fatigue-Inertia (FI)”, “Anger-Hostility (AH)” and “Vigor-Activity (VA).” A total negative mood score was calculated by subtracting the vigor scale from the sum of the remaining subscales. The scale showed good validity in related studies (Tang et al., 2007; Xu et al., 2015). The Chinese version revised by Wang also displays a high degree of validity with a Cronbach’s alpha level of 0.7∼0.9 in subscales among Chinese participants (Wang and Lin, 2000). Cronbach’s alpha for POMS-SF in this study was 0.90.

### Data Analysis

Descriptive analyses, *t*-test and Chi-square test were used to examine the group difference of basic characteristics. Linear mixed models were performed to account for the training effects. We fitted mixed-effects regression models for continuous variables (CPT RT, Stroop RT, VA, and so on; effect size reported in Cohen’s d) using baseline and 4th, 8th, and 12th week data. We tested the training effect between the two groups, controlling for sex, age and the baseline value of the outcome of interest. The mixed-effects models contain both fixed and random effects, with the fixed effects modeling treatment differences and random effects accounting for intra-subject variability. The regression models were conducted to explore the relationship between the outcome of mindfulness on emotion/cognition and between the outcome of emotion on cognition. Sex and baseline measure of each outcome measure were used as covariates in regression analyses. All analyses were implemented in SPSS.20.

Missing data were determined to be missing completely at random (MCAR) and suitable for using mixed-effects models analysis. We decided not to impute missing data to avoid introduction of extra bias to the model.

## Results

### Demographic and Functional Characteristics Between Two Groups

The MBT group (*n =* 24, age = 24.13 ± 5.11) consisted of 18 females (75%) and 6 males. The control group (*n =* 24, age = 24.25 ± 5.17) consisted of 16 females (66.7%) and 8 males. Demographic or baseline characteristics did not differ between the two groups [age (*t =* 0.08, *p =* 0.933), sex (χ^2^ = 0.40, *p* = 0.525), CPT hit rate baseline (*t =* 0.78, *p =* 0.970), CPT RT baseline (*t =* 0.04, *p =* 0.441), Stroop RT baseline (*t =* 0.44, *p =* 0.659), Stroop accuracy baseline (*t =* 1.41, *p =* 0.166), VA baseline (*t =* 0.26, *p =* 0.800), Negative emotion baseline (*t =* 0.63, *p =* 0.532), MAAS baseline (*t =* 1.17, *p =* 0.250) (see Table 1).

**TABLE 1:** Demographic and functional characteristics between two groups.

**Baseline demographic characteristics**	**MBT group (*N =* 24) *M ± SD***	**Control group (*N =* 24) *M ± SD***	***t /*χ^2^**	***p***
Female	18 (75%)	16 (66.7%)	0.40	0.525
Age	24.13±5.11	24.25±5.17	0.08	0.933
CPT hit rate baseline	0.96±0.06	0.97±0.03	0.78	0.970
CPT RT baseline	421.05±79.17	420.27±65.81	0.04	0.441
Stroop RT baseline	971.41±355.63	1015.81±337.17	0.44	0.659
Stroop accuracy baseline	0.93±0.07	0.95±0.06	1.41	0.166
VA baseline	14.25±3.89	14.50±2.83	0.26	0.800
Negative emotion baseline	59.08±14.23	56.29±16.43	0.63	0.532
MAAS baseline	56.67±14.49	60.96±10.76	1.17	0.250

*MBT, mindfulness-based training; CPT, the continuous performance test; VA, vigor-activity; MAAS, mindful attention awareness scale.*

### MBTs’ Effect on Cognition and Emotion

**FIGURE 2:**
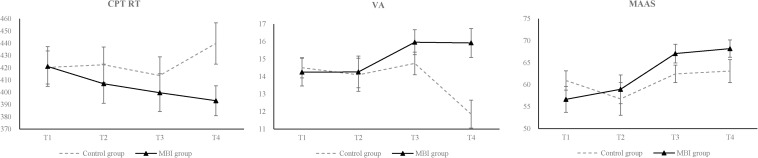
Comparison of cognition, emotion and mindfulness level during 12 weeks for the MBT and control group. Significance was found in The Continuous Performance Test reaction time (CPT RT), Vigor-Activity (VA) and Mindful Attention Awareness Scale score (MAAS). Error bars indicate 1 *SD*.

Table 2 presents results from the mixed models analyses. There were interactions between time and group in CPT RT [*F*(127.59) = 3.66, *p =* 0.014], VA [*F*(127.98) = 4.8, *p =* 0.003] and MAAS [*F*(127.68) = 3.08, *p =* 0.03], indicating that participants assigned to MBT group had a significantly greater reduction in CPT reaction time, significantly greater improvement in positive emotion and in MAAS than those assigned to control group (see Figure 2). Main effect of group for VA [*F*(43.54) = 5.3, *p =* 0.026] is significant, for CPT RT [*F*(44.24) = 3.86, *p =* 0.056] and MAAS [*F*(44.62) = 3.65, *p =* 0.063] were marginal significant (see Table 2). There were two main effects of time, for Stroop RT and MMAS.

**TABLE 2:** Mixed model analyses with between-group effect sizes (*N* = 48).

**Outcome measures**	**Treatment group**		**Time**		**Treatment group x time**		**T_2_**			**T_3_**			**T_4_**		
	***F***	***p***	***F***	***p***	***F***	***p***	**Adjusted mean difference (95% CI)**		**ES**	**Adjusted mean difference (95% CI)**		**ES**	**Adjusted mean difference (95% CI)**		**ES**
CPT_RT	*F*(44.24) = 3.86	0.056	*F*(127.48) = 1.24	0.299	*F*(127.59) = 3.66	0.014	9.42	(−34.83, 53.68)	0.22	10.88	(−35.1, 56.85)	0.2	46.78	(5.45, 88.1)	0.72
CPT hit rate	*F*(40.27) = 0.5	0.484	*F*(122.93) = 2.49	0.064	*F*(123.03) = 0.41	0.745	0.02	(−0.01, 0.04)	0.42	0.01	(−0.02, 0.04)	0.2	0	(−0.02, 0.03)	0.09
Stroop RT	*F*(43.2) = 0.38	0.541	*F*(126.79) = 11.55	<0.001	*F*(126.89) = 0.25	0.86	39.44	(−142.03, 220.91)	0.14	−21.7	(−132.45, 89.05)	0.12	83.93	(−41.31, 209.17)	0.42
Stroop accuracy	*F*(44.26) = 0.79	0.38	*F*(129.22) = 0.83	0.479	*F*(129.38) = 1.89	0.134	−0.03	(−0.09, 0.03)	0.31	−0.02	(−0.05, 0.01)	0.36	−0.03	(−0.08, 0.01)	0.48
VA	*F*(43.54) = 5.3	0.026	*F*(127.83) = 2.34	0.077	*F*(127.98) = 4.8	0.003	−0.16	(−2.81, 2.49)	0.04	−1.21	(−3.18, 0.77)	0.38	−4.07	(−6.41, −1.72)	1.08
Negative emotion	*F*(43.86) = 0.56	0.458	*F*(127.51) = 0.64	0.592	*F*(127.7) = 0.78	0.506	−1.65	(−14.39, 11.1)	0.08	2.05	(−8.43, 12.53)	0.12	4.23	(−5.15, 13.6)	0.28
MAAS	*F*(44.62) = 3.65	0.063	*F*(127.54) = 8.51	<0.001	*F*(127.68) = 3.08	0.03	−2.21	(−12.16, 7.75)	0.14	−4.63	(−10.61, 1.34)	0.48	−5.11	(−11.59, 1.37)	0.49

*ES, Between-group effect size (Cohen’s d); T_2_, 4th week; T_3_, 8th week; T_4_, 12th week; CPT RT, The continuous performance test reaction time; VA, vigor-activity.*

### The Effect of Mindfulness Level Predicted Change on Cognition and Emotion

Regression model 1 and 7 were conducted to explore the effect of group on cognition at T_3_ and T_4_. Regression model 2, 5, 8 11 were conducted to explore the effect of group on mindfulness level at T_2_ and T_3_. Regression model 3 and 9 were conducted to explore the effect of mindfulness level at T_n_ and Group in predicting cognition at T_n__+__1_ (1 < *n* ≤ 3). Regression model 4 and 10 were conducted to explore the effect of group on positive emotion at T_3_ and T_4_. Regression model 6 and 12 were conducted to explore the effect of mindfulness level at T_n_ and Group in predicting positive emotion at T_n__+__1_ (1 < *n* ≤ 3).

Regression model 1 was designed to estimate the effect of Group in predicting CPT RT T_3_. The regression coefficient of group in this model was −11.44, *p =* 0.541, *F*(3) = 7.60, *R^2^*= 0.33, *p <* 0.001 (see Table 3).

**TABLE 3:** Regression analysis showing the extent to MAAS predicted change on the outcome measures.

**Model**	**Dependent variable**	**Independent variable**	***B***	***SE***	***t***	***p_1_***	***F***	**df**	***R^2^***	***p_2_***
Model 1	CPT RT T_3_	Group	–11.44	18.54	–0.62	0.541	7.60	3	0.33	<0.001
		Constant	165.73	53.63	3.09	0.004				
Model 2	MAAS T_2_	Group	3.00	4.74	0.63	0.530	2.22	3	0.08	0.101
		Constant	40.23	11.78	3.41	0.002				
Model 3	CPT RT T_3_	MAAS T_2_	1.33	0.57	2.31	0.027	6.99	4	0.37	<0.001
		Group	–16.92	17.73	–0.95	0.346				
		Constant	85.96	65.02	1.32	0.195				
Model 4	VA T_3_	Group	1.23	0.97	1.27	0.210	1.63	3	0.04	0.198
		Constant	11.20	2.14	5.24	0.000				
Model 5	MAAS T_2_	Group	3.00	4.74	0.63	0.530	2.22	3	0.08	0.101
		Constant	40.23	11.78	3.41	0.002				
Model 6	VA T_3_	MAAS T_2_	–0.08	0.03	–2.77	0.009	3.41	4	0.19	0.018
		Group	1.54	0.90	1.71	0.095				
		Constant	16.80	2.80	6.01	0.000				
Model 7	CPT RT T_4_	Group	–46.87	17.17	–2.73	0.009	9.08	3	0.37	<0.001
		Constant	223.01	50.57	4.41	0.000				
Model 8	MAAS T_3_	Group	5.66	2.71	2.09	0.043	4.64	3	0.20	0.007
		Constant	41.48	6.75	6.15	0.000				
Model 9	CPT RT T_4_	MAAS T_3_	–0.18	1.00	–0.18	0.857	6.28	4	0.35	<0.001
		Group	–44.14	18.76	–2.35	0.024				
		Constant	228.54	67.29	3.40	0.002				
Model 10	VA T_4_	Group	3.97	1.17	3.39	0.002	4.62	3	0.20	0.007
		Constant	8.79	2.63	3.34	0.002				
Model 11	MAAS T_3_	Group	5.66	2.71	2.09	0.043	4.64	3	0.20	0.007
		Constant	41.48	6.75	6.15	0.000				
Model 12	VA T_4_	MAAS T_3_	0.10	0.06	1.60	0.118	4.27	4	0.24	0.006
		Group	3.72	1.23	3.02	0.005				
		Constant	3.20	4.29	0.75	0.460				

*Model 1, using Group as a predictor for CPT RT T_3_; Model 2 and Model 5, using Group as a predictor for MAAS T_2_; Model 3, using MAAS T_2_ and Group as predictors for CPT RT T_3_; Model 4, using Group as a predictor for VA T_3_; Model 6, using MAAS T_2_ and Group as predictors for VA T_3_; Model 7, using Group as a predictor for CPT RT T_4_; Model 8 and Model 11, using Group as a predictor for MAAS T_3_; Model 9, using MAAS T_3_ and Group as predictors for CPT RT T_4_; Model 10, using Group as a predictor for VA T_4_; Model 12, using MAAS T_3_ and Group as predictors for VA T_4_; Sex and baseline measure of each outcome measure used as covariates in all analyses. *p*_1_, significance of the coefficient; p_2_, significance of the regression model. MAAS, mindful attention awareness scale; CPT RT, The continuous performance test reaction time; VA, vigor-activity; T2, 4th week; T3, 8th week; T4, 12th week.*

Regression model 2 and 5 was designed to estimate the effect of Group in predicting MAAS T_2_. The regression coefficient of group in this model was 3.00, *p =* 0.53, *F*(3) = 2.22, *R*^2^ = 0.08, *p =* 0.101.

Regression model 3 was designed to estimate the effects of MAAS T_2_ and Group in predicting CPT RT T_3_. The regression coefficient of MAAS T_2_ in this model was 1.33, *p =* 0.027, of group in this model was −16.92, *p =* 0.346, *F*(4) = 6.99, *R^2^*= 0.37, *p <* 0.001, suggesting that the MAAS at T_2_ could significantly predict the CPT RT at T_3_.

Regression model 4 was designed to estimate the effect of Group in predicting VA T_3_. The regression coefficient of group in this model was 1.23, *p =* 0.210, *F*(3) = 1.63, *R*^2^ = 0.04, *p =* 0.198.

Regression model 6 was designed to estimate the effects of MAAS T_2_ and Group in predicting VA T_3_. The regression coefficient of MAAS T2 in this model was −0.08, *p =* 0.009, of group in this model was 1.54, *p =* 0.095, *F*(4) = 3.41, *R*^2^ = 0.19, *p =* 0.018, suggesting MAAS at T_2_ could significantly predict the VA at T_3_.

### The Effect of Positive Emotion Predicted Change on Cognition

Regression model 13 and model 16 were conducted to explore the effect of mindfulness level at T_n_ and Group in predicting cognition at T_n__+__2_ (1 < *n* ≤ 2). Regression model 14 and 17 were conducted to explore the effect of mindfulness level at T_n_ and Group in predicting positive emotion at T_n__+__1_ (1 < *n* ≤ 2). Regression model 15 and 18 were conducted to explore the effect of mindfulness level at T_n_ and positive emotion at T_n__+__1_ in predicting cognition at T_n__+__2_ (1 < *n* ≤ 2).

Regression model 13 was designed to estimate the effect of MAAS T1 and group in predicting CPT RT T_3_. The regression coefficient of MAAS T1 in this model was 0.07, *p =* 0.93, *F*(4) = 5.55, *R^2^*= 0.31, *p =* 0.001 (see Table 4).

**TABLE 4:** Regression analysis showing the extent to positive emotion predicted change on cognition.

**Model**	**Dependent variable**	**Independent variable**	***B***	***SE***	***t***	***p_1_***	***F***	**df**	***R^2^***	***p_2_***
Model 13	CPT RT T_3_	MAAS T_1_	0.07	0.76	0.09	0.93	5.55	4	0.31	0.001
		Group	–11.33	18.83	–0.60	0.551				
		Constant	162.29	66.89	2.43	0.020				
Model 14	VA T_2_	MAAS T_1_	0.03	0.05	0.65	0.519	1.06	4	0.01	0.387
		Group	0.18	1.31	0.14	0.889				
		Constant	13.81	3.75	3.68	0.001				
Model 15	CPT RT T_3_	VA T_2_	4.88	2.21	2.21	0.034	5.35	5	0.35	<0.001
		MAAS T_1_	–0.24	0.74	–0.32	0.751				
		Group	–14.72	18.07	–0.81	0.421				
		Constant	114.30	71.90	1.59	0.121				
Model 16	CPT RT T_4_	MAAS T_2_	1.00	0.57	1.76	0.087	7.23	4	0.39	<0.001
		Group	–47.41	17.81	–2.66	0.012				
		Constant	155.28	66.40	2.34	0.025				
Model 17	VA T_3_	MAAS T_2_	–0.08	0.03	–2.77	0.009	3.41	4	0.19	0.018
		Group	1.54	0.90	1.71	0.095				
		Constant	16.80	2.80	6.01	0.000				
Model 18	CPT RT T_4_	VA T_3_	–0.62	3.08	–0.20	0.841	5.63	5	0.37	<0.001
		MAAS T_2_	0.95	0.63	1.50	0.143				
		Group	–46.36	18.80	–2.47	0.019				
		Constant	166.25	86.53	1.92	0.063				

*Model 13, using MAAS T_1_ as a predictor for CPT RT T_3_; Model 14, using MAAS T_1_ as a predictor for VA T_2_; Model 15, using MAAS T_1_ and VA T_2_ as predictors for CPT RT T_3_; Model 16, using MAAS T_2_ as a predictor for CPT RT T_4_; Model 17, using MAAS T_2_ as a predictor for VA T_3_; Model 18, using MAAS T_2_ and VA T_3_ as predictors for CPT RT T_4_; Sex and baseline measure of each outcome measure used as covariates in all analyses. *p*_1_, significance of the coefficient; p_2_, significance of the regression model. MAAS, mindful attention awareness scale; CPT RT, The continuous performance test reaction time; VA, vigor-activity; T2, 4th week; T3, 8th week; T4, 12th week.*

Regression model 14 was designed to estimate the effect of MAAS T_1_ and group in predicting VA T_2_. The regression coefficient of MAAS T_1_ in this model was 0.03, *p =* 0.519, *F*(4) = 1.06, *R*^2^ = 0.01, *p =* 0.387.

Regression model 15 was designed to estimate the effects of MAAS T_1_, VA T_2_ and Group in predicting CPT RT T_3_. The regression coefficient of VA T_2_ in this model was 4.88, *p =* 0.034, of MAAS T_1_ in this model was −0.24, *p =* 0.751, *F*(5) = 5.35, *R^2^*= 0.35, *p =* 0.001, suggesting that the VA at T_2_ could significantly predict the CPT RT at T_3._

## Discussion

Based on eastern philosophy, mindfulness is becoming very popular for individuals seeking mental health and well-being in western countries. Although MBT has shown consistent efficacy for many disorders in western population, less attention has been given to the possible benefits that mindfulness may have in Non-clinical population, especially in China, one of the biggest countries in the eastern world.

This research mainly demonstrates that (a) a 12-week MBT can effectively improve sustained attention and positive emotion in Chinese Non-clinical population, (b) mindfulness training may take effect through the potential pathway of influencing cognition and emotion through the improvement in mindfulness level; (c) mindfulness training may take effect through the potential pathway of influencing cognition through the improvement in emotion.

The effect of MBT on cognition improvement is consistent with previous studies (Davidson et al., 1976; Jha et al., 2007; Schmertz et al., 2009; Zeidan et al., 2010; Lee and Orsillo, 2014; Morone et al., 2016). Meditation training might explain participants’ improved ability to focus, since meditation instructs to focus attention on breath, body parts, sounds, and Non-judgmentally come back to the object of focus when noticing mind-wandering.

In this study, the MBT was able to significantly improve participant’s positive emotion. This was consistent with previous research showing that control groups experienced significant drops in vigor and elevated levels of negative emotions compared to MBSR participants (Rosenzweig et al., 2003). Mindfulness practice may act as protective factor against the increased fatigue associated with repeated measures, helping participants to cultivate decreased emotional resistance and reactivity to present feelings, open and curious attitudes toward present-moment experience, and disengagement from rumination, a cognitive process shown to increase work-related fatigue (Deyo et al., 2009; Campbell et al., 2012).

The present findings of significant improvement in positive emotions and sustained attention in Non-clinical population offers new avenues for the application of mindfulness. The effects of MBT on individuals suffering from a variety of illnesses, like anxiety (Goldin and Gross, 2010; Mankus et al., 2013; Würtzen et al., 2013), depression (Deyo et al., 2009; Würtzen et al., 2013), chronic diseases (Bohlmeijer et al., 2010) and even cancer (Würtzen et al., 2013) have been demonstrated. In regard to Non-clinical individuals without obvious illnesses, however, this study’s findings bring two contributions. Firstly, mental health well-being rates may benefit from bringing clinical and Non-clinical population together in MBT, while both of them would benefit from MBT. In China, psychotherapy is underutilized because of stigma (Brown et al., 2010; Yap and Jorm, 2011), and treatment-seeking rates are less than 6% for common mental disorders (i.e., mood and anxiety disorders) (Charlson et al., 2016). Thus, mixing Non-clinical and clinical population in MBT could increase mental health seeking behaviors by reducing stigma. Secondly, stress is a pervasive issue in modern society and has become a global public health problem (Romas, 2009). Application of MBT might take effect in Non-clinical population as a tool for stress reduction, improvement of psychotherapy quality and prevention of mental disorders. Thus, MBT could serve as a preventative therapy and reduce stress that leads to unproductive rumination that consumes energy (Trapnell and Campbell, 1999), and can adversely affect emotion (Allen et al., 2014) and cognition (Liston et al., 2009; Wu et al., 2014).

Besides, we explored the potential pathway of mindfulness and found that mindfulness training may take effect through the potential pathway of influencing cognition and emotion through the improvement in mindfulness level. It was in accordance with the previous studies (Gu et al., 2015). This finding provides evidence for exploring the mechanism of mindfulness training, which stressing the importance of mindfulness level measurement. Further research could be refined to determine which component of mindfulness work on these outcomes. It might be the mindfulness attitude (such as acceptance) or mindfulness related practice (such as body scanning). And, at what point the component begins to take effect could also be studied to further explore the mechanisms of mindfulness.

The results indicated that mindfulness training may take effect through the potential pathway of influencing cognition through the improvement in emotion, which was a complement to the knowledge that rare studies have provided evidence for the assumption that mindfulness may impact cognition through emotion. Positive emotion at T_2_ could significantly predict sustained attention at T_3_, but this relationship did not exist in positive emotion at T_3_ and sustained attention at T_4_. There are three possible reasons. Firstly, the reduced size of the sample is one limitation. Secondly, the lack of follow-up measurement making it difficult to assess if anything is happened and maintained after the intervention. Thirdly, experimental paradigms and cognitive indicators used in this study may not be sensitive enough to indicated a stabilization trend, and the experimental paradigms and cognitive indicators need to be improved in the future. Nonetheless, the results of this research partially support our hypothesis that mindfulness training may take effect through the potential pathway of influencing cognition through the improvement in emotion. Recent studies begin to focus on the relationship between outcomes of mindfulness training, but there is no direct evidence to support. Now we could see the light of dawn on the horizon. Based on these results of the pilot study, we could improve the study design and measurements in the following research, and further verify our experimental hypothesis in larger sample. The potential pathway of mindfulness through the relationship between cognition and emotion establishes a base from which further studies could continue investigating the mechanisms for mindfulness. Future research should extend this small body of evidence for emotion as mechanisms.

The evidence of effectiveness of MBT was supported in Chinese Non-clinical populations. Mindfulness originated from eastern philosophy, but less attention has been given to Chinese populations while China is the most populous eastern country. The effectiveness of MBT in Chinese populations can further extend the application of mindfulness. Besides, there are many similarities between Chinese culture and the concept of mindfulness. Further studies are necessary to explore the difference of MBT effect between eastern and western populations.

While the results of the study are promising, a couple limitations should nonetheless be kept in mind. Firstly, a wait-list condition would might be more effective to future studies. Because the control group was not active, these results could be partly explained by an effect related to the group intervention setting (i.e., social support, shared positive emotions) and not the mindfulness training itself. Secondly, there was no follow-up measurement after the intervention program, making it difficult to assess if anything is maintained after the intervention and therefore, making it difficult to make interpretative hypothesis related to a potential prevention effect of the program on medium or long term. Thirdly, regarding validity, the size of the participants’ sample was small and the ratio male/female, reduced. Thus, further research should explore the significance of a larger sample size. Fourthly, trainees’ mental health was assessed as “Non-clinical” without use of formal scales for mental health disorders before the study. Future studies could use scales to ensure participants’ condition. Finally, no formal tracking of trainee’s personal practice occurred. Yet, participants reported in sessions practicing every week. Thus, this study’s results open avenues of research regarding the feasibility and effectiveness of mindfulness-based trainings adopting voluntary participation. Besides, mindfulness serving as an effective self-help method (Lever et al., 2014), more researchers are turning their attention to web-based mindfulness trainings (Cavanagh et al., 2013; Dimidjian et al., 2014), which involve reduced monitoring of personal practice. Further research exploring the effectiveness of MBT in China could have beneficial implications for the spread of MBT among Chinese populations.

## Data Availability

The data sets generated for this study are available on request to the corresponding author.

## Ethics Statement

This study was carried out in accordance with the recommendations of Institutional Review Board of Zhejiang University. All subjects gave written informed consent in accordance with the Declaration of Helsinki.

## Author Contributions

TZ and SC contributed to the conception and design of the study. TZ organized the database. TZ and JX performed the statistical analysis. TZ wrote the first draft of the manuscript. JX, AM, and SC wrote sections of the manuscript. WW designed the program. YJ assisted with the data collection. All authors contributed to the manuscript revision, read and approved the submitted version.

## Conflict of Interest Statement

AM was involved in the creation and/or facilitation of the MBT evaluated in this study. The remaining authors declare that the research was conducted in the absence of any commercial or financial relationships that could be construed as a potential conflict of interest.

## Appendix

**TABLE:** Description of 12-Week Mindfulness Training.

**Week**	**Topic**	**Content**
1	Mindfulness Introduction	Introductions, training schedule; Exercise on recognizing auto-pilot and automatic thinking with introduction to awareness of breath, body, and surroundings; Mindfulness definition and usual misconceptions
2	Exploring Here and Now	Introduction to Mindful Logs and Gratitude Journal; Mindful Eating: Raisin meditation; Intro to Body Scan Meditation (BSM)
3	Mindful Yoga	Introduction to Mindful Yoga, 40’ Mindful Yoga Session; Sharing of experience with BSM
4	9 Mindfulness attitudes	15-min BSM; Introduction to mindfulness’s 9 attitudes; 7-min MM on Emotions
5	Managing Stress and Difficult Situations	20 min MM on emotions; Introduction of RAIN Method; The Guest House Poem, from Rumi; 3-min Breathing Space MM
6	Mindful Communication and Thought Management	15-min Body and Sound MM; Introduction to observation of and Non-identification to thoughts; Introduction to identifying and overcoming obstacles to mindful communication; 8-min MM on Sounds and Thoughts
7	Loving-kindness and Self-compassion	8-min MM on Sounds and Thoughts; Introduction to Loving-kindness and usual misconceptions; 10-min Loving-Kindness Meditation; Try being kind to oneself in difficult situation.
8	Living wholeheartedly	8-min MM on Sounds and Thoughts; Watching of “The Power of Vulnerability” from Brene Brown; Discussion about mindfulness, vulnerability, compassion, connection and courage to face oneself and others authentically; 10-min Mindful Walking Meditation
9	Tying concepts together	15-min Body and Breath Meditation; Review of all mindfulness concepts and attitudes, mind management meditation; 15-min Mindful walking
10	6-h Silent Retreat in one of Hangzhou’s Yoga Centers	Sitting meditation; Mindfulness Yoga; Body scan meditation; Mindful walking; Gratitude meditation
11	Using mindfulness at Work	15 min Mindful Walking; Open discussion on how mindfulness could be used in careers as a mean of self-care and improved working environment
12	Keeping up with mindfulness training and conclusion	Sharing of resources and tips for continued mindfulness practice; STOP method; Body scan meditation

*Encouraged Weekly Practice (EWP) in each week.*

## References

[B1] Allen A. P. Kennedy P. J. Cryan J. F. Dinan T. G. Clarke G. (2014). Biological and psychological markers of stress in humans: focus on the trier social stress test. *Neurosci. Biobehav. Rev.* 38 94–124. 10.1016/j.neubiorev.2013.11.005 24239854

[B2] Alsubaie M. Abbott R. Dunn B. Dickens C. Keil T. F. Henley W. (2017). Mechanisms of action in mindfulness-based cognitive therapy (MBCT) and mindfulness-based stress reduction (MBSR) in people with physical and/or psychological conditions: a systematic review. *Clin. Psychol. Rev.* 55 74–91. 10.1016/j.cpr.2017.04.008 28501707

[B3] Am V. D. V. Kuyken W. Wattar U. Crane C. Pallesen K. J. Dahlgaard J. (2015). A systematic review of mechanisms of change in mindfulness-based cognitive therapy in the treatment of recurrent major depressive disorder. *Clin. Psychol. Rev.* 37 26–39. 10.1016/j.cpr.2015.02.001 25748559

[B4] Arch J. J. Craske M. G. (2006). Mechanisms of mindfulness: emotion regulation following a focused breathing induction. *Behav. Res. Ther.* 44 1849–1858. 10.1016/j.brat.2005.12.007 16460668

[B5] Armony J. L. Dolan R. J. (2002). Modulation of spatial attention by fear-conditioned stimuli: an event-related fMRI study. *Neuropsychologia* 40 817–826. 10.1016/s0028-3932(01)00178-6 11900732

[B6] Bailey N. W. Bridgman T. K. Marx W. Fitzgerald P. B. (2016). Asthma and mindfulness: an increase in mindfulness as the mechanism of action behind breathing retraining techniques? *Mindfulness* 7 1–7.

[B7] Berger I. Cassuto H. (2014). The effect of environmental distractors incorporation into a CPT on sustained attention and ADHD diagnosis among adolescents. *J. Neurosci. Methods* 222 62–68. 10.1016/j.jneumeth.2013.10.012 24211249

[B8] Bohlmeijer E. Prenger R. Taal E. Cuijpers P. (2010). The effects of mindfulness-based stress reduction therapy on mental health of adults with a chronic medical disease: a meta-analysis. *J. Psychosom. Res.* 68 539–544. 10.1016/j.jpsychores.2009.10.005 20488270

[B9] Brake C. A. Sauer-Zavala S. Boswell J. F. Gallagher M. W. Farchione T. J. Barlow D. H. (2016). Mindfulness-based exposure strategies as a transdiagnostic mechanism of change: an exploratory alternating treatment design. *Behav. Ther.* 47 225–238. 10.1016/j.beth.2015.10.008 26956654 PMC8177505

[B10] Brittlebank A. D. Scott J. Williams J. M. Ferrier I. N. (1993). Autobiographical memory in depression: state or trait marker? *Br. J. Psychiatry* 162 118–121. 10.1192/bjp.162.1.118 8425125

[B11] Brown C. Conner K. O. Copeland V. C. Grote N. Beach S. Battista D. (2010). Depression stigma, race, and treatment seeking behavior and attitudes. *J. Community Psychol.* 38 350–368. 10.1002/jcop.20368 21274407 PMC3026177

[B12] Campbell T. S. Labelle L. E. Bacon S. L. Faris P. Carlson L. E. (2012). Impact of mindfulness-based stress reduction (MBSR) on attention, rumination and resting blood pressure in women with cancer: a waitlist-controlled study. *J. Behav. Med.* 35 262–271. 10.1007/s10865-011-9357-1 21667281

[B13] Carlson L. E. Brown K. W. (2005). Validation of the mindful attention awareness scale in a cancer population. *J. Psychosom. Res.* 58 29–33. 10.1016/j.jpsychores.2004.04.366 15771867

[B14] Cavanagh K. Strauss C. Cicconi F. Griffiths N. Wyper A. Jones F. (2013). A randomised controlled trial of a brief online mindfulness-based intervention. *Behav. Res. Ther.* 51 573–578. 10.1016/j.brat.2013.06.003 23872699

[B15] Chambers D. A. Schauenstein K. (2000). Mindful immunology: neuroimmunomodulation. *Trends Immunol.* 21 168–170. 10.1016/s0167-5699(99)01577-7 10798848

[B16] Chambers R. Gullone E. Allen N. B. (2009). Mindful emotion regulation: an integrative review. *Clin. Psychol. Rev.* 29 560–572. 10.1016/j.cpr.2009.06.005 19632752

[B17] Chambers R. Gullone E. Hassed C. Knight W. Garvin T. Allen N. (2015). Mindful emotion regulation predicts recovery in depressed youth. *Mindfulness* 6 523–534. 10.1007/s12671-014-0284-4

[B18] Chambers R. Lo B. C. Y. Allen N. B. (2008). The Impact of intensive mindfulness training on attentional control, cognitive style, and affect. *Cogn. Ther. Res.* 32 303–322. 10.1007/s10608-007-9119-0

[B19] Charlson F. J. Baxter A. J. Cheng H. G. Shidhaye R. Whiteford H. A. (2016). The burden of mental, neurological, and substance use disorders in china and india: a systematic analysis of community representative epidemiological studies. *Lancet* 388 376–389. 10.1016/S0140-6736(16)30590-6 27209143

[B20] Chen S. Cui H. Zhou R. Jia Y. (2012). Revision of MIndful attention awareness scale(MAAS). *Chinese J. Clin. Psychol.* 20 148–151.

[B21] Chiesa A. Calati R. Serretti A. (2011). Does mindfulness training improve cognitive abilities? a systematic review of neuropsychological findings. *Clin. Psychol. Rev.* 31 449–464. 10.1016/j.cpr.2010.11.003 21183265

[B22] Chiesa A. Serretti A. (2009). Mindfulness-based stress reduction for stress management in healthy people: a review and meta-analysis. *J. Altern. Complem. Med.* 15 593–600. 10.1089/acm.2008.0495 19432513

[B23] Chiesa A. Serretti A. (2011). Mindfulness based cognitive therapy for psychiatric disorders: a systematic review and meta-analysis. *Psychiatry Res.* 187 441–453. 10.1016/j.psychres.2010.08.011 20846726

[B24] Cornblatt B. A. Risch N. J. Faris G. Friedman D. Erlenmeyer-Kimling L. (1988). The continuous performance test, identical pairs version (CPT-IP): i. new findings about sustained attention in normal families. *Psychiatry Res.* 26 223–238. 10.1016/0165-1781(88)90076-5 3237915

[B25] Davidson R. J. Goleman D. J. Schwartz G. E. (1976). Attentional and affective concomitants of meditation: a cross-sectional study. *J. Abnorm. Psychol.* 85 235–238. 10.1037//0021-843x.85.2.235 1254784

[B26] Deyo M. Wilson K. A. Ong J. Koopman C. (2009). Mindfulness and rumination: does mindfulness training lead to reductions in the ruminative thinking associated with depression? *Explore* 5 265–271. 10.1016/j.explore.2009.06.005 19733812

[B27] Diamond A. Lee K. (2011). Interventions shown to aid executive function development in children 4 to 12 years old. *Science* 333 959–964. 10.1126/science.1204529 21852486 PMC3159917

[B28] Dimidjian S. Beck A. Felder J. N. Boggs J. M. Gallop R. Segal Z. V. (2014). Web-based mindfulness-based cognitive therapy for reducing residual depressive symptoms: an open trial and quasi-experimental comparison to propensity score matched controls. *Behav. Res. Ther.* 63 83–90. 10.1016/j.brat.2014.09.004 25461782 PMC5714615

[B29] Dolan R. J. (2002). Emotion, cognition, and behavior. *Science* 298 1191–1194. 12424363 10.1126/science.1076358

[B30] Eslinger P. J. (1992). *The Amygdala: Neurobiological Aspects of Emotion, Memory, and Mental Dysfunction.* New York, NY: Wiley-Liss.

[B31] Goldin P. R. Gross J. J. (2010). Effects of mindfulness-based stress reduction (MBSR) on emotion regulation in social anxiety disorder. *Emotion* 10 83–91. 10.1037/a0018441 20141305 PMC4203918

[B32] Gong P. Liang S. Carlton E. J. Jiang Q. Wu J. Wang L. (2012). Urbanisation and health in china. *Lancet* 379 843–852. 22386037 10.1016/S0140-6736(11)61878-3 PMC3733467

[B33] Gu J. Strauss C. Bond R. Cavanagh K. (2015). How do mindfulness-based cognitive therapy and mindfulness-based stress reduction improve mental health and wellbeing? a systematic review and meta-analysis of mediation studies. *Clin. Psychol. Rev.* 37 1–12. 10.1016/j.cpr.2015.01.006 25689576

[B34] Hoge E. A. Bui E. Marques L. Metcalf C. A. Morris L. K. Robinaugh D. J. (2013). Randomized controlled trial of mindfulness meditation for generalized anxiety disorder: effects on anxiety and stress reactivity. *J. Clin. Psychiatry* 74 786–792. 10.4088/JCP.12m08083 23541163 PMC3772979

[B35] Hülsheger U. R. Alberts H. J. E. M. Feinholdt A. Lang J. W. B. (2013). Benefits of mindfulness at work: the role of mindfulness in emotion regulation, emotional exhaustion, and job satisfaction. *J. Appl. Psychol.* 98 310–325. 10.1037/a0031313 23276118

[B36] Jha A. P. Krompinger J. Baime M. J. (2007). Mindfulness training modifies subsystems of attention. *Cogn. Affect. Behav. Neurosci.* 7 109–119. 10.3758/cabn.7.2.109 17672382

[B37] Junwon K. Youngsik L. Doughyun H. Kyungjoon M. Dohyun K. Changwon L. (2015). The utility of quantitative electroencephalography and integrated visual and auditory continuous performance test as auxiliary tools for the attention deficit hyperactivity disorder diagnosis. *Clin. Neurophysiol.* 126 532–540. 10.1016/j.clinph.2014.06.034 25088931

[B38] Kabatzinn J. (2003). Mindfulness-based interventions in context: past, present, and future. *Clin. Psychol. Sci. Practice* 10 144–156. 10.1093/clipsy/bpg016

[B39] Klee S. H. Garfinkel B. D. (1983). The computerized continuous performance task: a new measure of inattention. *J. Abnorm. Child Psychol.* 11 487–495. 10.1007/bf00917077 6689172

[B40] Lee J. K. Orsillo S. M. (2014). Investigating cognitive flexibility as a potential mechanism of mindfulness in generalized anxiety disorder. *J. Behav. Ther. Exp. Psychiatry* 45 208–216. 10.1016/j.jbtep.2013.10.008 24239587

[B41] Lever T. B. Strauss C. Cavanagh K. Jones F. (2014). The effectiveness of self-help mindfulness-based cognitive therapy in a student sample: a randomised controlled trial. *Behav. Res. Ther.* 63 63–69. 10.1016/j.brat.2014.09.007 25302763

[B42] Lindsay E. K. Creswell J. D. (2017). Mechanisms of mindfulness training: monitor and acceptance theory (MAT). *Clin. Psychol. Rev.* 51 48–59. 10.1016/j.cpr.2016.10.011 27835764 PMC5195874

[B43] Liston C. Mcewen B. S. Casey B. J. (2009). Psychosocial stress reversibly disrupts prefrontal processing and attentional control. *Proc. Natl. Acad. Sci. U.S.A.* 106 912–917. 10.1073/pnas.0807041106 19139412 PMC2621252

[B44] Lutz A. Slagter H. A. Rawlings N. B. Francis A. D. Greischar L. L. Davidson R. J. (2009). Mental training enhances attentional stability: neural and behavioral evidence. *J. Neurosci.* 29 13418–13427. 10.1523/JNEUROSCI.1614-09.2009 19846729 PMC2789281

[B45] Lyvers M. Makin C. Toms E. Thorberg F. A. Samios C. (2014). Trait Mindfulness in relation to emotional self-regulation and executive function. *Mindfulness* 5 619–625. 10.1007/s12671-013-0213-y

[B46] Macaskill A. (2013). The mental health of university students in the united kingdom. *Br. J. Guid. Counc.* 41 426–441. 10.1080/03069885.2012.743110

[B47] Mankus A. M. Aldao A. Kerns C. Mayville E. W. Mennin D. S. (2013). Mindfulness and heart rate variability in individuals with high and low generalized anxiety symptoms. *Behav. Res. Ther.* 51 386–391. 10.1016/j.brat.2013.03.005 23639305

[B48] Manoj S. Rush S. E. (2014). Mindfulness-based stress reduction as a stress management intervention for healthy individuals: a systematic review. *J. Evid. Based Complem. Altern. Med.* 19 271–286. 10.1177/2156587214543143 25053754

[B49] Mendelson T. Greenberg M. T. Dariotis J. K. Gould L. F. Rhoades B. L. Leaf P. J. (2010). Feasibility and preliminary outcomes of a school-based mindfulness intervention for urban youth. *J. Abnorm. Child Psychol.* 38 985–994. 10.1007/s10802-010-9418-x 20440550

[B50] Ministry of Education of the People’s Republic of China (2018). *National Education Career Development Statistical Report in 2017.* Beijing: Ministry of Education of the People’s Republic of China.

[B51] Mogg K. Bradley B. P. De B. J. Painter M. (1997). Time course of attentional bias for threat information in non-clinical anxiety. *Behav. Res. Ther.* 35 297–303. 10.1016/s0005-7967(96)00109-x 9134784

[B52] Morone N. E. Rollman B. L. Moore C. G. Qin L. Weiner D. K. (2016). A Mind–Body program for older adults with chronic low back pain: results of a pilot study. *JAMA Int. Med.* 176 329–337. 10.1111/j.1526-4637.2009.00746.x 26903081 PMC6361386

[B53] Moynihan J. A. (2013). Mindfulness-based stress reduction for older adults: effects on executive function. *Neuropsychobiology* 68 34–43. 10.1159/000350949 23774986 PMC3831656

[B54] Napoli D. M. Krech P. R. Holley L. C. (2005). Mindfulness training for elementary school students. *J. Appl. School Psychol.* 21 99–125. 10.1300/j370v21n01_05

[B55] Ong J. C. Manber R. Segal Z. Xia Y. Shapiro S. Wyatt J. K. (2014). A randomized controlled trial of mindfulness meditation for chronic insomnia. *Sleep* 37 1553–1563. 10.5665/sleep.4010 25142566 PMC4153063

[B56] Romas J. A. (2009). Practical Stress Management.

[B57] Rosenzweig S. Reibel D. K. Greeson J. M. Brainard G. C. Hojat M. (2003). Mindfulness-based stress reduction lowers psychological distress in medical students. *Teach. Learn. Med.* 15 88–92. 10.1207/s15328015tlm1502_03 12708065

[B58] Schmertz S. K. Anderson P. L. Robins D. L. (2009). The relation between self-report mindfulness and performance on tasks of sustained attention. *J. Psychopathol. Behav. Assess.* 31 60–66. 10.1007/s10862-008-9086-0

[B59] Shapiro S. L. Brown K. W. Biegel G. M. (2007). Teaching self-care to caregivers: effects of mindfulness-based stress reduction on the mental health of therapists in training. *Train. Educ. Prof. Psychol.* 1 105–115. 10.1037/1931-3918.1.2.105

[B60] Tang Y. Y. Ma Y. Fan Y. Feng H. Wang J. Feng S. (2009). Central and autonomic nervous system interaction is altered by short-term meditation. *Proc. Natl. Acad. Sci.U.S.A.* 106 8865–8870. 10.1073/pnas.0904031106 19451642 PMC2690030

[B61] Tang Y. Y. Ma Y. Wang J. Fan Y. Feng S. Lu Q. (2007). Short-Term meditation training improves attention and self-regulation. *Proc.Natl. Acad. Sci.U.S.A.* 104 17152–17156. 10.1073/pnas.0707678104 17940025 PMC2040428

[B62] Tang Y. Y. Posner M. I. (2015). The neuroscience of mindfulness meditation. *Nat. Rev. Neurosci.* 16 213–225. 10.1038/nrn3916 25783612

[B63] Tang Y. Y. Yang L. Leve L. D. Harold G. T. (2012). Improving executive function and its neurobiological mechanisms through a mindfulness-based intervention: advances within the field of developmental neuroscience. *Child Dev. Perspect.* 6 361–366. 25419230 10.1111/j.1750-8606.2012.00250.x PMC4238887

[B64] Trapnell P. D. Campbell J. D. (1999). Private self-consciousness and the five-factor model of personality: distinguishing rumination from reflection. *J. Pers. Soc. Psychol.* 76 284–304. 10.1037//0022-3514.76.2.284 10074710

[B65] Wang J. Lin W. (2000). POMS for use in China. *Acta. Psychologica Sinica* 32 110–114.

[B66] Wheeler M. S. Arnkoff D. B. Glass C. R. (2017). The neuroscience of mindfulness: how mindfulness alters the brain and facilitates emotion regulation. *Mindfulness* 8 1471–1478. 10.3389/fnhum.2018.00448 30483083 PMC6243128

[B67] Williams J. M. Teasdale J. D. Segal Z. V. Soulsby J. (2000). Mindfulness-based cognitive therapy reduces overgeneral autobiographical memory in formerly depressed patients. *J. Abnorm. Psychol.* 109 150–155. 10.1037//0021-843x.109.1.150 10740947

[B68] Wu J. Yuan Y. Duan H. Qin S. Buchanan T. W. Kan Z. (2014). Long-term academic stress increases the late component of error processing: An ERP study. *Biol. Psychol.* 99 77–82. 10.1016/j.biopsycho.2014.03.002 24657630

[B69] Würtzen H. Dalton S. O. Elsass P. Sumbundu A. D. Steding-Jensen M. Karlsen R. V. (2013). Mindfulness significantly reduces self-reported levels of anxiety and depression: results of a randomised controlled trial among 336 Danish women treated for stage I-III breast cancer. *Eur. J. Cancer* 49:1365. 10.1016/j.ejca.2012.10.030 23265707

[B70] Xu W. Wang Y. Z. Liu X. H. (2015). Effectiveness of 8-week mindfulness training improving negative emotions. *Chinese Mental Health J.* 29 497–502

[B71] Yap M. B. H. Jorm A. F. (2011). The influence of stigma on young people’s help-seeking intentions and beliefs about the helpfulness of various sources of help. *Soc. Psychiatry Psychiatr. Epidemiol.* 46 1257–1265. 10.1007/s00127-010-0300-5 20938638

[B72] Zeidan F. Johnson S. K. Diamond B. J. David Z. Goolkasian P. (2010). Mindfulness meditation improves cognition: evidence of brief mental training. *Conscious. Cogn.* 19 597–605. 10.1016/j.concog.2010.03.014 20363650

[B73] Zhong B. L. Chan S. Liu T. B. Jin D. Hu C. Y. Hfk C. (2017). Mental health of the old- and new-generation migrant workers in China: who are at greater risk for psychological distress? *Oncotarget*. 8 59791–59799. 10.18632/oncotarget.15985 28938682 PMC5601778

